# Intussusception in Mosaic Trisomy 14

**DOI:** 10.14309/crj.0000000000001296

**Published:** 2024-03-04

**Authors:** Jeremy Skvarce, Arjun Chatterjee, Giselle Velez, Ram Gurajala, Jeffrey Schwartz, Manuel B. Braga-Neto

**Affiliations:** 1 Department of Internal Medicine, Cleveland Clinic Foundation, Cleveland, OH; 2 Department of Hospital Medicine, Cleveland Clinic Foundation, Cleveland, OH; 3 Department of Interventional Radiology, Cleveland Clinic Foundation, Cleveland, OH; 4 Department of Gastroenterology and Hepatology, Digestive Disease Institute, Cleveland Clinic, Cleveland, OH

**Keywords:** Trisomy 14, intussusception, abdominal pain, constipation

## Abstract

Mosaic trisomy 14 is exceptionally rare and was first described in the 1970s with fewer than 100 known liveborn individuals. Information about complications and the natural history of the disease is rare, especially in adult patients. This case illustrates an adult patient with severe functional limitations from mosaic trisomy 14 who presented with abdominal pain and failure to thrive and was subsequently found to have intussusception and severe chronic constipation, which was successfully treated conservatively.

## INTRODUCTION

Trisomy 14 is a rare chromosomal disorder associated with a high rate of infant mortality requiring either a mosaic genotype or unbalanced Robertsonian translocation for survival into adulthood.^1^ It disproportionately affects females (3:1) with common morphologic features including a broad nose, a short neck, congenital heart disease, growth delay, and developmental delay.^1-3^ The spectrum of functional status and survival is broad, and the scarcity of data concerning complications and prognosis creates significant challenges in clinical decision-making and offering guidance to patients' families. To the best of our knowledge, no previous episodes of intussusception have been reported within the limited available data in this patient population, and we hope this case can better inform care of patients with this rare condition.^4,5^

## CASE REPORT

A 26-year-old non-verbal woman with mosaic trisomy 14 complicated by chronic constipation was admitted to our hospital for failure to thrive because of poor oral intake. Our patient was nonverbal, making investigation into her poor oral intake difficult with pertinent history obtained from her mother. At baseline, the patient was tolerating multiple food servings every day, despite severe chronic constipation, with bowel movements spaced up to 3 weeks apart at times. Her home bowel regimen included nightly bisacodyl suppositories, polyethylene glycol, and senna. Three months before presentation, the patient was noted to have a loss of appetite associated with significant weight loss of up to 30% of baseline weight. She initially presented at an outside facility, where she was noted to be uncomfortable appearing, although afebrile and hemodynamically stable. On physical examination, her abdomen was nondistended with normal bowel sounds, but she had left upper-quadrant tenderness. Laboratory findings on presentation included unremarkable complete blood count and comprehensive metabolic panel. She underwent an esophagogastroduodenoscopy with gastric biopsy showing mild superficial chronic gastritis, with unremarkable esophageal and duodenal biopsies exonerating eosinophilic esophagitis and celiac disease, respectively. She subsequently represented to our institution for a second opinion.

**Figure 1.**
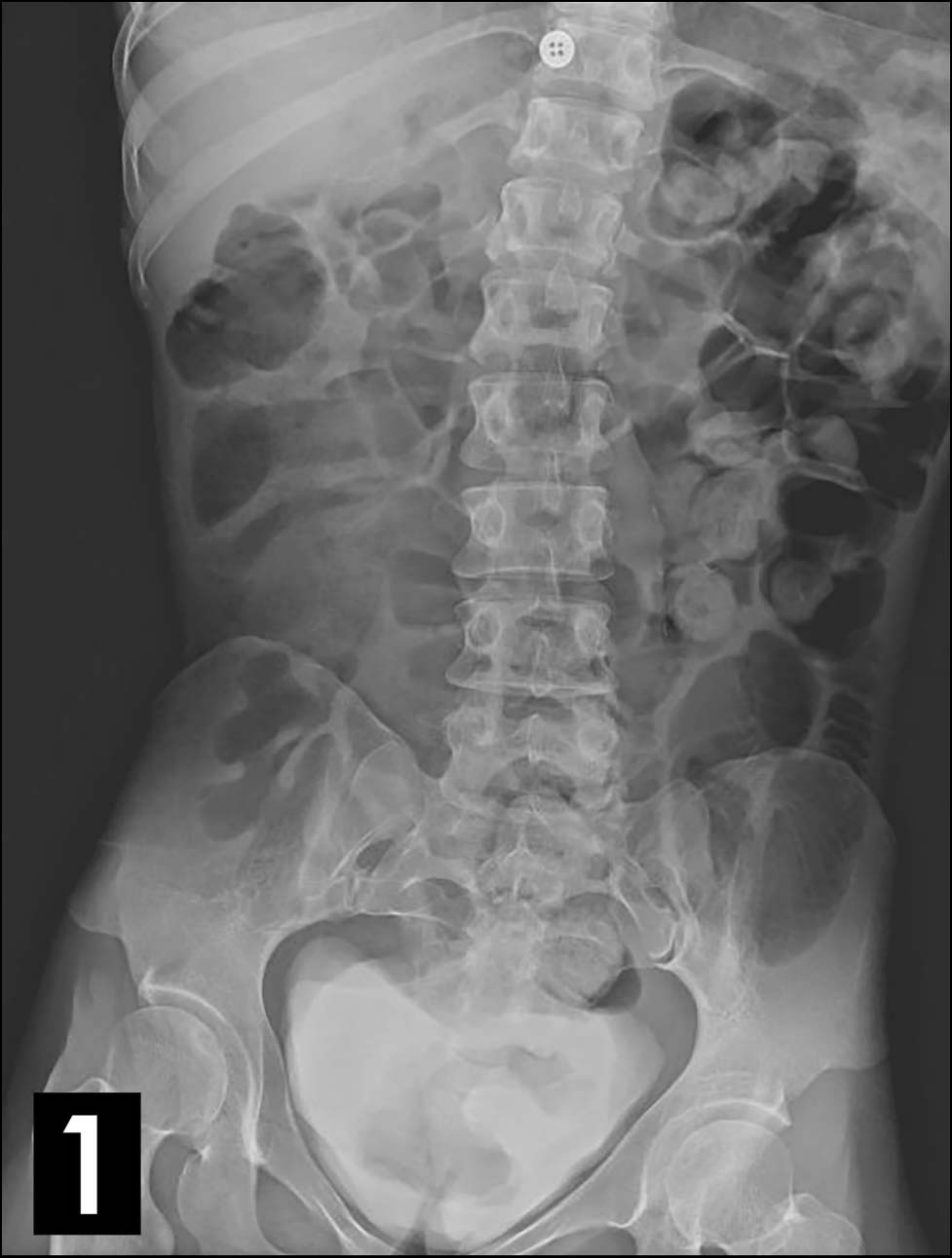
Abdominal X-ray demonstrated mildly dilated small bowel loops with gas throughout the large bowel.

**Figure 2.**
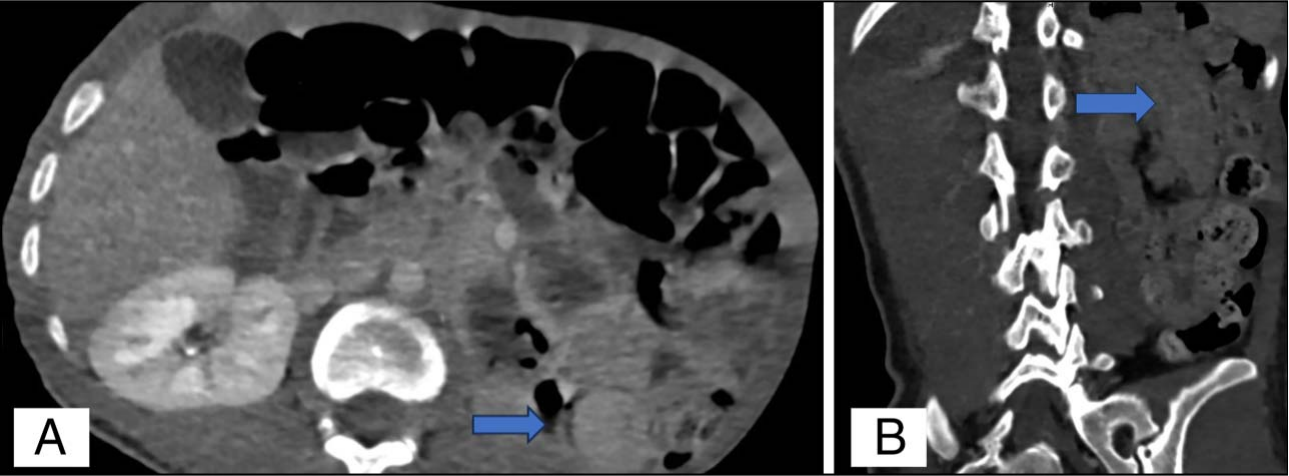
Abdominal and pelvic computed tomography axial (A) and coronal (B) sections demonstrating a circular focus of small bowel (jejunum, 5.5 cm) abutting the posterior abdominal wall, with a “target sign” appearance (blue arrow) within the posterior left upper quadrant suggestive of intussusception.

On arrival, abdominal x-ray was notable for significant stool burden with mildly dilated small bowel loops with gas throughout the normal caliber large bowel (Figure 1). She subsequently underwent abdominal and pelvic computed tomography (CT), which revealed a circular focus of small bowel (jejunum, 5.5 cm) abutting the posterior abdominal wall, with a “target sign” appearance within the posterior left upper quadrant suggestive of intussusception (Figure 2). No obvious vasculature etiology was elucidated, and no other foci of intussusception were seen.

**Figure 3.**
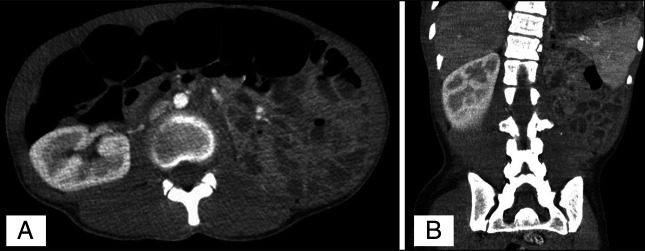
Abdominal and pelvic computed tomography axial (A) and coronal (B) demonstrating resolution of small bowel intussusception.

Reassuringly, the patient continued to show no clinical signs of obstruction, and her follow-up abdominal examination remained unchanged. Patient's bowel regimen was optimized by adding polyethylene glycol and senna, which improved her oral intake and abdominal tenderness on physical examination. Interval CT imaging showed resolution of the intussusception (Figure 3). Although laparotomy with bowel resection had been considered as a contingency should patient progress to a full bowel obstruction with an acute abdomen, no invasive intervention was required.

She was discharged with proper caregiver education, and outpatient follow-up was planned for both evaluation of possible small bowel malignancy as possible lead point through video capsule endoscopy as well as for further underlying dysmotility workup. Unfortunately, the patient was lost to follow-up.

## DISCUSSION

Our case highlights the challenges in managing complications associated with mosaic trisomy 14. This nonverbal patient with mosaic trisomy 14 presented with abdominal pain and significant malnutrition secondary to decreased oral intake. Further investigation revealed transient intussusception in the setting of profound stool burden, with resolution of symptoms after aggressive bowel regimen. Although difficult to ascertain definitive causality in this case, the significance of the stool burden distal to the intussusception suggests that mechanical obstruction caused by the intussusception was not responsible for the patient's constipation. Rather, we hypothesize that severe constipation was the major risk factor for intussusception in this case, as has been previously reported.^6^ Initially, it was believed that the patient may require surgical intervention if her mechanical obstruction did not resolve; however, conservative therapy proved to be successful.

Intussusception occurs primarily at points of transition within the gastrointestinal tract, such as the junctions between mobile and fixed segments of bowel or between different segments. These locations include enteroenteric, colocolic, ileocolic (involving the terminal ileum and ascending colon), and ileocecal (involving the ileocecal valve, which acts as the leading point of the intussusception).^7–9^ Intussusception in pediatric patients frequently lacks a known cause, whereas in adults, approximately 90% of cases have a pathologic lead point, such as carcinomas, polyps, Meckel's diverticulum, colonic diverticulum, strictures, or benign neoplasms.^7^ Adult intussusception accounts for only 5% of all intussusception cases and contributes to 1%–5% of adult intestinal obstructions.^8^

Adult intussusception presents diversely; unlike classic pediatric symptoms, adults may have nonspecific indicators such as nausea, vomiting, bleeding, altered bowel habits, and distension.^8,9^

Diagnosing adult intussusception is challenging because of diverse symptoms and imaging findings. Initial approaches involve abdominal X-rays, whereas ultrasonography reveals characteristic features such as the “target,” “doughnut,” or “pseudokidney” signs.^7,10^ Abdominal CT scans are particularly sensitive and can illustrate a homogeneous “target” or “sausage”-shaped soft-tissue mass with layering effects.^7,11^

In adults, arriving at a preoperative diagnosis of intussusception is frequently complex because of its vague symptoms, resulting in instances of overlooked or delayed identification, often necessitating surgical treatment.^7,11^ Unlike in pediatric cases, attempting preoperative reduction with barium or air is not a definitive treatment for adults because of the incidence malignant lead point of intussusception nearing 50% of all cases.^12^

Our patient's physiological and developmental characteristics more closely resembled those of a pediatric patient, making their case a unique challenge to navigate; however, although trisomy 14 remains very rare, these challenges exist across a spectrum of developmental disorders, which result in similarly impaired patient communicative and functional status. With only a limited number of documented instances of trisomy 14 within this particular age bracket, the inherent disease progression in the patient remained unclear. Nevertheless, conservative approach successfully resolved her intussusception. This case contributes to our understanding of potential complications associated with mosaic trisomy 14 and provides valuable guidance to future clinicians dealing with intussusception in this rare population.

## DISCLOSURES

Author contributions: J. Skvarce and A. Chatterjee: analysis, interpretation, and drafting. A. Chatterjee, G. Velez, and J. Schwartz: drafting and reviewing. R. Gurajala: acquisition of images. MB Braga Neto: accountable for the accuracy and ethical conduct of the case report and is the article guarantor.

Financial disclosure: None to report.

Informed consent was obtained for this case report.
